# Factors influencing wild chimpanzee (*Pan troglodytes verus*) relative abundance in an agriculture-swamp matrix outside protected areas

**DOI:** 10.1371/journal.pone.0215545

**Published:** 2019-05-16

**Authors:** Rosa M. Garriga, Ignasi Marco, Encarna Casas-Díaz, Pelayo Acevedo, Bala Amarasekaran, Luna Cuadrado, Tatyana Humle

**Affiliations:** 1 Servei d’Ecopatologia de Fauna Salvatge, Facultat de Veterinària, Universitat Autònoma de Barcelona, Barcelona, Spain; 2 Tacugama Chimpanzee Sanctuary, Freetown, Sierra Leone; 3 Departament de Sanitat i Anatomia Animals, Facultat de Veterinària, Universitat Autònoma de Barcelona, Barcelona, Spain; 4 Instituto de Investigación en Recursos Cinegéticos, Ciudad Real, Spain; 5 Durrell Institute of Conservation and Ecology, School of Anthropology and Conservation, University of Kent, Canterbury, United Kingdom; Sichuan University, CHINA

## Abstract

Human population growth and anthropogenic activities are exacerbating pressures on biodiversity globally. Land conversion is aggravating habitat fragmentation and non-human primates are increasingly compelled to live in forest-agricultural mosaics. In Sierra Leone, more than half of the wild chimpanzee population (*Pan troglodytes verus*) occurs outside protected areas and competes for resources with farmers. Our study area, in the Moyamba district in south-western Sierra Leone, is practically devoid of forest and is dominated by cultivated and fallow fields, swamps and mangroves. In this region, traditional slash-and-burn agriculture modifies annually the landscape, sparing swamps and mangroves and semi-domesticated oil palms (*Elaeis guineensis*). This study aimed to explore ecological and anthropogenic factors influencing chimpanzee relative abundance across this highly degraded and human-impacted landscape. Between 2015 and 2016, we deployed 24 camera traps systematically across 27 1.25x1.25 km grid cells. Cameras were operational over a period of 8 months. We used binomial iCAR models to examine to what extent anthropogenic (roads, settlements, abandoned settlements and human presence) and habitat variables (swamps, farmland and mangroves) shape chimpanzee relative abundance. The best model explained 43.16% of the variation with distance to roads and swamps emerging as the best predictors of chimpanzee relative abundance. Our results suggest that chimpanzees avoid roads and prefer to maintain proximity to swamps. There was no significant effect of settlements, abandoned settlements, mangroves or human presence. It appears that chimpanzees do not avoid areas frequented by people; although, our findings suggest temporal avoidance between the two species. We highlight the importance of studying chimpanzee populations living in anthropogenic habitats like agricultural-swamp matrixes to better understand factors influencing their distribution and inform conservation planning outside protected areas.

## Introduction

It is estimated that approximately 60% of all world's non-human primates (hereafter primates) are threatened with extinction and the main threats to their survival are habitat loss and fragmentation due to rapid human population growth and land conversion for agriculture [1–3]. Many primate species are having to live in forest-agricultural mosaics and in close proximity to people [4]. West Africa has one of the most fragmented tropical forest landscapes in the world due to high levels of deforestation [5]. Some animal species are nevertheless able to adjust to these changes and survive and even flourish in human altered conditions [6]. Some primates also show a certain degree of flexibility and can adapt their dietary, socioecological behaviours to these human altered landscapes [7,8]. In recent years, there has been a growing interest in studying primates, and especially chimpanzees (*Pan troglodytes*), living in human-modified habitats to understand how they behave and survive in degraded landscapes and which conservation strategies may be best suited for the species in areas outside protected areas [8].

Chimpanzee populations across Africa are declining due to habitat loss, poaching and disease [9]. They can occur in a variety of habitats from moist lowland to mountain forests, swamp forests and woodland savannas [10], but rapid human population growth and agricultural expansion into forested areas have compelled chimpanzee populations to survive in forest-agricultural mosaics [9]. Chimpanzees are able to adjust their behaviour to some level of human disturbances like agriculture, selective logging and/or low levels of hunting [7,11,12]. Behavioural adjustments include dietary [13–15] and socioecological adaptations [13,16–18], as well as behavioural responses to novel risks such as roads [19,20], and the presence of people, such as researchers and farmers [21].

Anthropogenic landscapes in which primates co-exist with people consist of a complex mosaic of forest and agricultural habitat types intermixed with roads and settlements often bordering protected areas [8]. Studies in Uganda have estimated similar densities of chimpanzees in an agroforestry landscape bordering the Budongo Forest Reserve, highlighting the importance of such anthropogenic habitat for the conservation of primates. However, the absence of hunting and the proximity to this large protected forest may explain primates’ persistence in these fragmented forest blocks [22]. Nevertheless, many chimpanzee populations also occur in highly degraded anthropogenic landscapes away from protected areas and with only small remnant forest fragments [23,24]. Typically, such forest patches are riverine, flooded or swamp forests usually unsuitable for agriculture but important habitats for wildlife [21]. Swamps can sometimes act as a barrier to chimpanzee dispersal such as in the Kahuzi-Biega National Park in the Democratic Republic of Congo [25]. However, in other regions, swamp forests and mangroves can also act as critical habitat for great apes [26–31] for feeding, nesting and as a refuge from hunters.

Road development in Africa is directly related to human expansion [32,33] and extractive industries such as mining and logging [34,35]. Such infrastructure development can exacerbate habitat loss and fragmentation, as well as wildlife mortality, including that of chimpanzees, directly via road kills [36] or indirectly by facilitating hunting and the bushmeat trade [33,37]. In a nationwide study conducted in Gabon, Vanthomme [35] revealed that chimpanzee relative abundance is negatively affected by the presence of main roads (>15 m wide; tar and laterite coated). However, this study failed to find any significant relationship with proximity to secondary roads (typically sand coated and in poorer condition) and human settlements. In Guinea-Bissau, chimpanzees preferred to build their nests farther away from roads (main and secondary grouped together) [38]. Another study in a forest concession in Gabon revealed that neither main roads nor settlements influenced chimpanzee distribution [39]. These differing findings may be linked to differences in hunting pressure, road width and traffic intensity, as well as people presence and behaviour towards chimpanzees. Duvall [40] also highlighted the value of abandoned settlements for chimpanzees, as these areas provide highly nutritious food resources such as bananas (*Musa sp*.), oranges (*Citrus aurantifolia*), and mangos (*Mangifera indica*) long after abandonment. However, hunters and local people may also frequent such areas, potentially acting as deterrents to wildlife frequentation [40].

The IUCN status of the western subspecies of chimpanzee, *P*. *t*. *verus*, has been upgraded recently to Critically Endangered [41]. Sierra Leone harbours the third largest chimpanzee population in West Africa with more than half living outside protected areas [23,42]. It is estimated that since 1975, the country has lost 36% of its forest and woodland habitats [43]. ‘Farm-bush’, the degraded secondary forest growth that follows slash-and-burn agriculture, is increasingly the most dominant vegetation type in Sierra Leone [44]. In the late 80s, Davies [45] had already noted that chimpanzees in Sierra Leone frequented cultivated areas across the entire country. However, only a few studies in Sierra Leone have since been published exploring how chimpanzees are able to persist in such agricultural matrixes [7,23]. Chimpanzees face serious threats in Sierra Leone, including habitat loss, hunting, and retaliation as a result of competition with people for resources [23,46]. The persistence of chimpanzees in agricultural matrixes and their co-existence with farmers has been documented in other parts of Africa [24], but still remains largely understudied. Such persistence has mostly been attributed to human tolerance linked to religious and cultural taboos [47,48], access to highly nutritious cultivars [49] and the importance of semi-domesticated oil palms (*Elaeis guineensis*) for nesting and food (e.g. Bossou, Guinea [50]; Guinea-Bissau [51,52].

Although chimpanzees occur across a wide range of habitat types, few studies have focused on chimpanzees inhabiting highly degraded landscapes practically devoid of forest and dominated by farmland and swamps, as well as human presence and infrastructures such as roads and settlements. Our study aimed to fill this gap using camera trapping technology in an agricultural-swamp matrix in the Moyamba district in south-western Sierra Leone. We examined habitat preferences and the influence of human disturbance at a fine spatial scale on wild chimpanzee relative abundance across the landscape. Our specific objectives were: 1) to determine the relative population abundance across the study area, and 2) to explore spatial variations in relative abundance patterns in relation to anthropogenic (roads, settlements, abandoned settlements and human presence) and environmental (swamps, farmland and mangroves) features. Considering the extent to which chimpanzees occur outside protected areas in Sierra Leone, this study aimed to contribute valuable insights into factors affecting persistence of chimpanzees in highly deforested landscapes to better inform current and future conservation efforts. Assuming that chimpanzees in our landscape seek to avoid as much as possible human presence and associated infrastructures such as roads and active settlements, we hypothesized the spatial distribution of chimpanzees’ relative abundance will be mostly influenced by a) the presence of people and roads, b) swamps and mangroves which are less frequented by people than farmland, and c) abandoned than active settlements.

## Material and methods

### Study area

The study area, called Lawana, is located in the coastal plains in the Moyamba district in the south-western Sierra Leone in West Africa. It covers approximately 91 km^2^ in the chiefdoms of Bumpeh and Kagboro (12°46’31”W and 7°59’55”N) with elevation ranges of 1 to 37 m above sea level. The habitat is characterised by active and fallow farms at various stages of regrowth, swamps and mangroves intermixed with settlements and unpaved roads with a forest cover of <1% as estimated during this study (see details below). Semi-domesticated oil palms are abundant and are the most frequently encountered tree species across this agricultural matrix. The climate is tropical with a dry season which runs from November to May [46]. The total human population is rural with an average density of 51.5 hab/km^2^ for Bumpeh and 55.9 hab/km^2^ for Kagboro chiefdoms [53]. Subsistence farming is the main human activity in the study area. Farmers cultivate seasonal crops like rice (*Oryza spp*.), cassava (*Manihot esculenta)*, sesame (*Sesamum* sp.) and sorghum (*Sorghum bicolor*) using slash-and-burn intercropping practices. Previous investigations in the study area confirmed the presence of chimpanzees through semi-structured interviews as well as direct observations [23,46].

### Camera trap survey

This study was approved by the University of Kent’s Animal Welfare Ethics Review Board and permission to conduct the research was granted by the National Protected Areas Authority, an autonomous entity under the Ministry of Agriculture, Forestry and Food Security of Sierra Leone. We also secured verbal permission from village chiefs and farmers to set cameras on their land, assuring them that any data on human presence captured via camera traps would be treated anonymously and used solely to estimate the extent of overlap in habitat use between chimpanzees and people.

**Fig 1:**
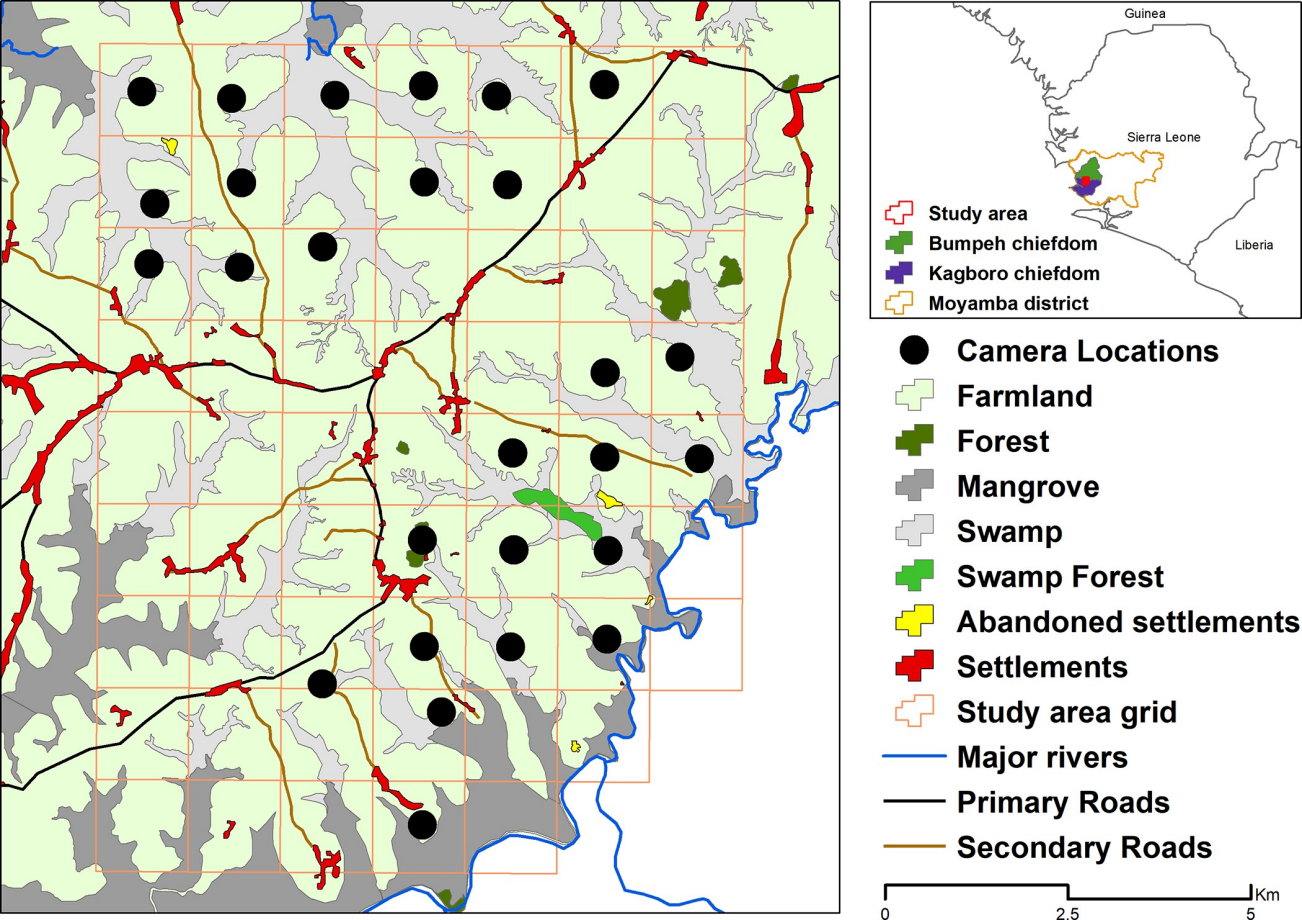
Study area showing the 27 camera traps locations and the habitat types in the district of Moyamba in Sierra Leone. Upper right map shows the location of the study area in the chiefdoms of Bumpeh and Kagboro. Source of land cover information: National Protected Areas Authority in Sierra Leone.

We conducted our camera trap survey during 8 months between April 2016 and May 2017 divided into three time periods during the dry season months: April to May 2016, November 2016 to February 2017 and February to May 2017. We used ARCGIS 10.3 (ESRI, Redlands, USA) to design a sampling grid with cell sizes of 1.25x1.25 km^2^. The chimpanzee home range in the study area was unknown, and we defined our grid cell size based on approximate average minimum day range from Basabose [25], Bates and Byrne [54] and unpublished data from released chimpanzees equipped with store-on-board tracking collars in a savanna dominated landscape (Humle, unpub. data). We set one camera within each block for each period. Due to limitations in the number of cameras, sampling was focused in the areas with expected presence of chimpanzees according to confirmations from farmers across villages in the study landscape. In each grid, cameras were adjusted at approximately 1m high to oil palms, bushes or tree trunks at sites with evidence of animal activity within 100 m of the centre of each grid. Vegetation 5 m in front of the camera was brushed to minimise the risk of false triggers and cameras were secured with python locks. In some instances, we had to shift a camera’s placement or remove it altogether because of ongoing slash-and-burn agricultural activities. Therefore, a few locations were not sampled during the entire sampling period. For analytical purposes, we selected cameras that were deployed in the same location at least during 2 of the 3 time periods. We used 24 infra-red digital camera traps Reconyx HC500, HC600 and PC800 (Reconyx Inc., Holmen, WI, USA) and all were programmed with the same settings, i.e. high sensitivity, three consecutive pictures and no delay, resolution of 3.1 MP, 24h operational, with date and time stamp and infra-red mode. In total, we surveyed 27 grids of which 17 locations were surveyed for three periods and 10 for two periods (Fig 1).

### Habitat classification

The habitat was classified manually using satellite imagery with a 30 m resolution (GeoEye, WorldView-2 and WorldView-3 satellites acquired between 2015 and 2017). The landscape is a mixture of habitat types, in which farmland is markedly predominant. Despite the area being relatively small, it is at the intersection of different satellite images for different time periods and the annual slash-and-burn farming practices consequently rendered it impossible to classify different farmland types (i.e. burnt land, new farm, young fallow and old fallow). Therefore, we classified all the farmland as one single type. Table 1 describes the habitats and vegetation types present in the study area. A large river, the Kagboro River, delimits the southern side of the study area and is assumed to act as a natural barrier to chimpanzee dispersal (Fig 1). Human settlements were divided in two categories, ‘small’ with fewer than 25 households (ranging from 0.12–2.9 hectares) and ‘large’ with more than 25 households (ranging from 3–38 hectares). Hamlets that were less than 200 m away from each other were considered as part of the same settlement, unless the presence of a swamp warranted their separate categorisation. This classification system was also corroborated via ground-truthing observations.

**Table 1:** Habitat types surface (and in percentage) in the study area.

Habitat type	Description	Total area (km^2^)	Percentage of the total area
Farmland	Includes: young fallow, mature fallow, cultivated land and burnt fields for cultivation	66.84	73.05%
Swamp	Dominated by raffia palms	15.75	17.22%
Mangrove	Dominated by mangrove shrubs	6.86	7.5%
Urban	Settlements	1.34	1.46%
Forest	Mature secondary regrowth of vegetation. 30+ years old with a closed canopy	0.36	0.39%
Swamp Forest	Forest which is inundated with freshwater, either permanently or seasonally	0.27	0.29%
Abandoned settlements	Areas in which there was a settlement in the past. No houses remain but fruit producing orchards persist.	0.08	0.09%
Total		**91.5**	**100%**

### Anthropogenic and environmental variables

Aside from the human camera trapping rate, all other anthropogenic predictor variables used in the analysis were based on the shortest Euclidean distance [55] between the camera trap and the road network, settlements, abandoned settlements, mangroves, swamps and farmland (Table 2). All predictors were based at the grid-level using the ‘*raster’* package [56] in the R software [57]. We used GIS Software ArcGIS 10.3 to calculate the percentage of habitat types in each grid and we created 5 categorical habitat variables depending on the dominant habitat types in each grid: farmland, swamps, mangroves and farmland/swamps and farmland/mangroves. These latter mixed categories were created when none of the habitat types in the grid was predominant. It must be noted, however, that these two categories were finally not included in the models since they were highly correlated to distance to swamps and mangroves.

**Table 2:** Description of the habitat and anthropogenic variables used as predictors in the analysis.

Type	Variables	Description	Measure
Anthropogenic Variables	Small settlements	Fewer than 25 households.	Distance from camera location to nearest feature (m)
	Large settlements	More than 25 households.	Distance from camera location to nearest feature (m)
	Roads	Includes all motor-able roads. All were unpaved.	Distance from camera location to nearest feature (m)
	Abandoned settlements	Areas in which there was a settlement in the past. No houses remain but fruit producing orchards persist.	Distance from camera location to nearest feature (m)
	All urbanised areas	Small and large settlements merged.	Distance from camera location to nearest feature (m)
	Human trapping rate	Comparing human and chimpanzee TR	Number of events x trap-days per camera location
Habitat variables	Farmland	Cultivated land active and fallow	Distance from camera location to nearest feature (m)
	Swamp	Uncultivated land where water and raffia palms dominate	Distance from camera location to nearest feature (m)
	Mangrove	A tidal swamp which is dominated by mangrove shrubs	Distance from camera location to nearest feature (m)

### Statistical modelling

Camera images were screened for species identification by RG and confirmed by LC, TH and another independent recorder. The images were analysed using the ZSL-CTAT open-access software developed at Zoological Society of London (ZSL) specifically to process images from camera trap arrays [58]. In this analysis, the software was set to score a new independent event (IE) when a sequence of images of a target species appeared more than 60 minutes after the previous images of that species [59]. We considered species trapping rates (TR) as proxies of relative abundance. TRs were calculated as the mean number of independent photographic events per trap day x 100, using cameras that operated for more than 75% of the survey period. We defined the sampling occasion as 5 consecutive days of monitoring. The same approach was used to derive trapping rates for both people and chimpanzees.

The chimpanzee home range in the study area was most probably greater than our grid cell size (1.25x1.25 km^2^) and therefore we expected some spatial autocorrelation in our data. To overcome this issue, we used the ‘hSDM.binomial.iCAR’ function of the ‘hSDM’ package [60] within the R statistical environment [57]. This function performs a logistic regression model (events versus occasions) in a hierarchical Bayesian framework accounting for spatial autocorrelation using an intrinsic conditional autoregressive (iCAR) model. iCAR assumes that the amount of events at one site depends on its amount on the neighbouring sites, in this case the eight cells around a target one. We also plotted the temporal activity pattern overlap between humans and chimpanzees using the ‘overlap’ R package [61].

## Results

**Fig 2:**
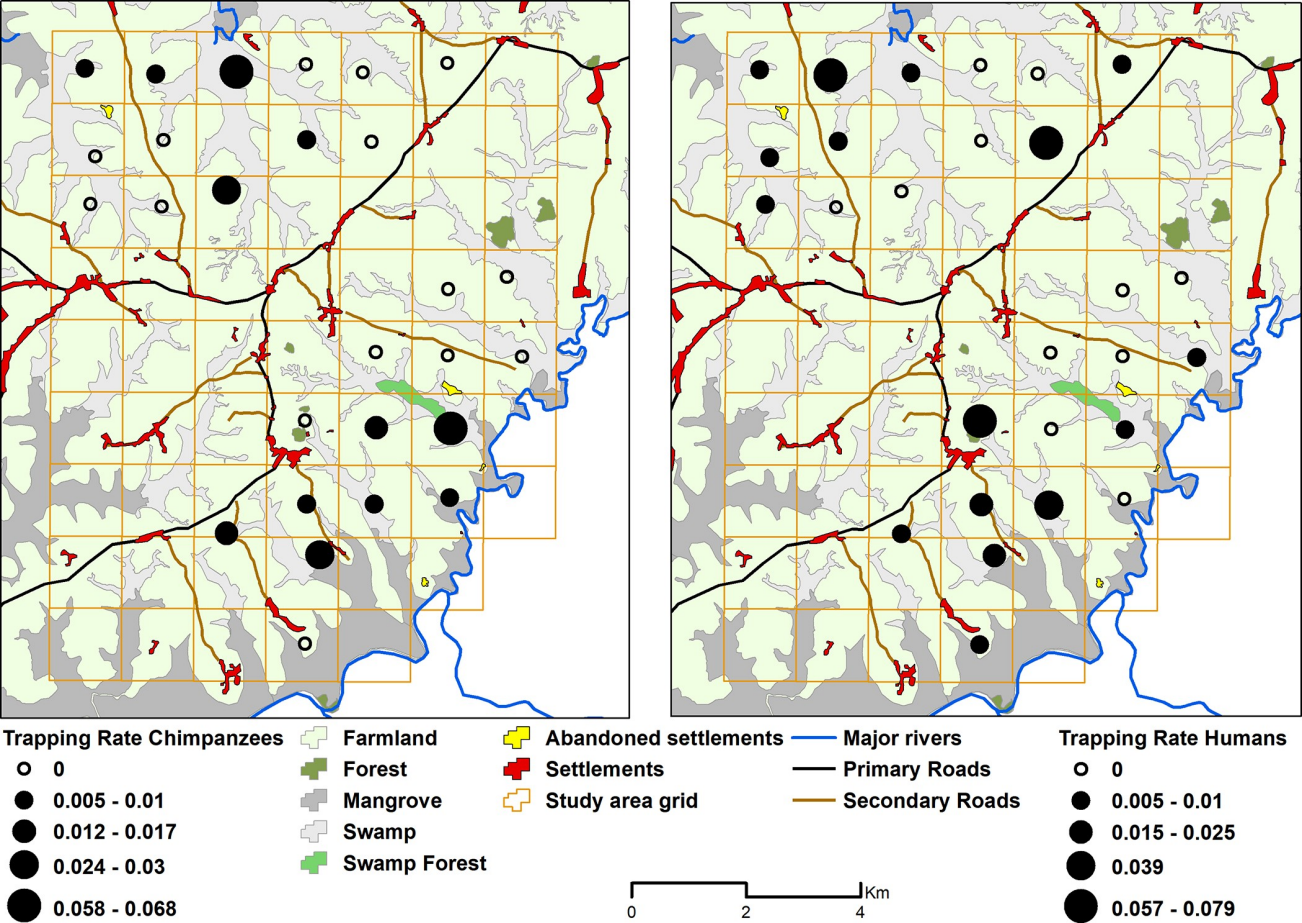
Chimpanzee and human trapping rates (number of independent events per trap-day) for each camera location. Source of land cover information: National Protected Areas Authority in Sierra Leone.

Cameras were operational for 4,763 trap-days with an average of 176 operational trap-days (range: 119–207 trap-days) per location. We recorded 44 chimpanzee IE in 12 locations (44.4% of the total locations) and 65 human IE in 16 locations (59.3% of the total locations) during the whole study period. Both species were photo-captured from the same location at 8 camera trapping sites (29.6% of the total locations). Chimpanzee and human TR for each camera location is shown in Fig 2.

We performed model selection using a forward stepwise procedure using deviance explained to select the final model (Table 3); stepwise stops when the addition of a new variable in the model did not significantly improve the explained deviance [62].

**Table 3:** Summary of the stepwise model selection procedure, based on the residual deviance, used to explain chimpanzees’ relative abundance.

Residual deviance	Model
96.39	Null model [M1]
63.21	M1 + iCAR [M2]
60.24	M2 + roads [M3]
54.79	M3 + swamps [final model]

**Fig 3:**
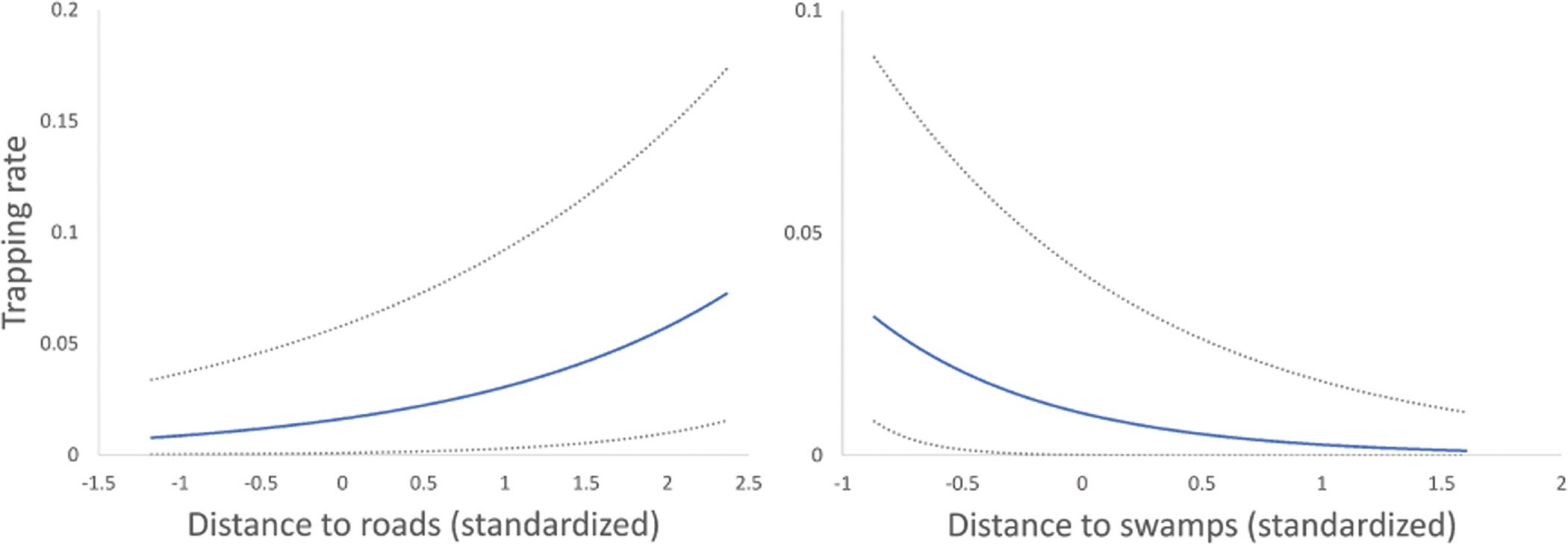
Statistically significant factors retained in the final binomial iCAR model explaining variations in chimpanzee trapping rate: a) distance to roads and b) distance to swamps. Plots show 95% credibility intervals.

Modelling showed that both anthropogenic and ecological features are relevant drivers of chimpanzee TR. However, only the distance to roads and the proximity to swamps served as good predictors of chimpanzee TR, yielding a best final model explaining 43.16% of the total deviance. The relevant amount of variability in trapping rates was explained by the iCAR component (Table 4). The best final model revealed that chimpanzees avoid roads and prefer to maintain proximity to swamps (Fig 3). The variables that were not included in the final model and failed to act as good predictors were human TR, distance to settlements and mangroves, and habitat type.

**Table 4:** Results of the binomial–iCAR final model examining the contribution of roads and swamps to chimpanzee trapping rates.

	Mean	SD Native	SE	Time series SE	Quantiles		
					2.5%	75%	97.5%
Intercept	-5.638	0.937	0.042	0.058	-7.774	-4.944	-4.184
Roads	0.748	0.399	0.018	0.019	0.034	1.011	1.505
Swamps	-2.830	1.572	0.070	0.101	-6.648	-1.712	-0.168
Vrho	6.716	2.076	0.093	0.093	2.290	8.482	9.940

Vrho: Spatial random effect variance; SD: Standard deviation; SE: Standard error of the mean.

**Fig 4:**
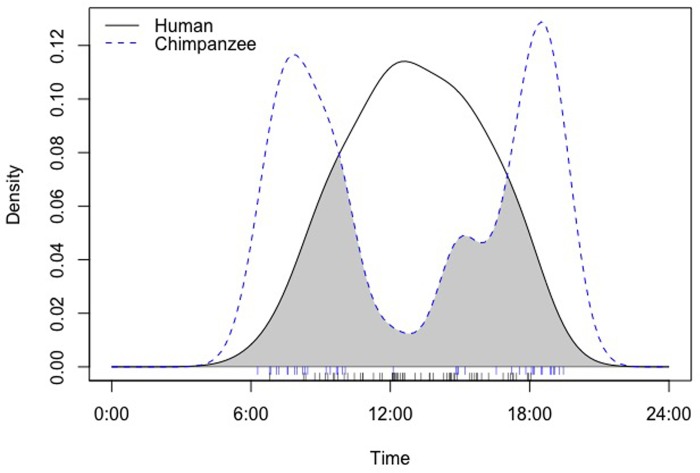
Plot showing the temporal overlap between human and chimpanzee activity patterns.

The temporal overlap index between human and chimpanzee activity patterns was not significant (Dhat = 0.52). The plot showed higher activity for humans during the central hours of the day and for chimpanzees during early and later hours of the day (Fig 4).

## Discussion

This study provides an assessment of the importance of habitat and anthropogenic variables on the relative abundance of chimpanzees across a predominantly farmland habitat at a fine spatial scale during dry season months. Our hierarchical Bayesian model accounting for spatial autocorrelation, revealed that roads negatively and swamps positively explained chimpanzee relative abundance across this landscape. Contrary to expectations, neither habitat (swamp, mangrove or farmland) nor the presence of active or abandoned settlements nor human presence influenced relative abundance of chimpanzees across our study landscape.

The negative influence of roads on chimpanzee relative abundance from our study corroborates large scale studies which have revealed reduced chimpanzee abundance near major roads in Sierra Leone [7] and in Gabon [35]. However, these studies also concluded that chimpanzee abundance was positively associated with secondary roads. In our study area, all roads could be considered secondary; these were indeed untarmacked, with variable frequency of use by vehicles, motorcycles and pedestrians. Such a finding is therefore critical when one thinks of the rapid expansion of road construction across Africa [63] and highlights the fact that roads can certainly impact chimpanzee distribution and abundance, even smaller secondary roads [38]. Chimpanzees living in fragmented habitats typically cross roads to move from one area to another in their home range and often approach human settlements [32,35,47,64]. Chimpanzee abundance and distribution might be negatively influenced by the proximity to roads and therefore the risks associated with the probability of encountering people. A recent study revealed that the distance from roads was indeed the best predictor of bonobo nest occurrence; however, this study argued that hunting of apes in proximity to the road rather than displacement of the bonobos, best explained this pattern [33,65]. Our study provides only insights during the dry season, November to May; it therefore, remains to be established whether reported findings persist during rainy season months, i.e. June to October, when fruit availability and habitat conditions, and hence chimpanzee behaviour, may differ.

In our model, settlements and human presence did not influence chimpanzees’ relative abundance, possibly due to human tolerance for chimpanzees [46] and low levels of hunting in our study area [7]. Therefore, at a spatial level, human presence did not impact chimpanzee presence. However, at a temporal level, chimpanzees tended to reduce their activity at midday when human activity was more prevalent, indicating a certain degree of temporal divergence in activity as an adaptation to human-impacted habitats. Such temporal avoidance has also been noted among Sebitoli chimpanzees in Kibale National Park, Uganda, where chimpanzees show some nocturnal activity patterns to avoid human presence [17,64]. A similar pattern of spatial overlap and temporal avoidance, with a shift towards cathemerality between people and wildlife has been reported in other animal species[66,67]. Although our study did not reveal increased cathemerality in our chimpanzee study population or spatial avoidance, it revealed a strong tendancy for temporal diurnal avoidance in areas frequented by both people and chimpanzees.

The study area is remote and there are no short-term plans by the government to improve road infrastructure; however, during our study, at least three footpaths leading to small settlements had been widened and cleared by the local people to allow for car passage. Moreover, in the last 5 years, there has been a boom in motorcycles becoming the most common means of local transportation. People use them as taxis and they can circulate at high speeds even along small footpaths, therefore increasing disturbance to wildlife and the risk of collision. The unpaved roads and the relatively low traffic levels in our study area probably explain why there have been no reported cases of chimpanzee road kills as in other parts of Africa such as Uganda [36]. Nevertheless, chimpanzees may adjust their behaviour when crossing roads such as looking right and left before and while crossing, crossing in small and more cohesive groupings, and increasing their waiting time, to reduce the danger posed by roads and avoid the risk of collision [19,20]. Similar adaptations could be occurring with the chimpanzees in our study area, but such adaptations if prevalent still fail to explain why they tended to avoid roads. Our findings suggest that we need to better understand the impact of roads on chimpanzee distribution and presence at a finer spatial scale, especially in light of the future road developments across their range.

Proximity to swamps was a good predictor of chimpanzee relative abundance in this landscape. Swamps represent the second largest habitat in the study area, while forest patches were extremely rare. However, remnant swamps and swamp forests are not cultivated by farmers and they might act as a refuge for chimpanzees and other wildlife possibly due to their greater inaccessibility as argued by Poulsen and Clark [27] in Northern Congo. Semi-domesticated oil palms are widely distributed across this landscape including inside swamp areas, offering chimpanzees a relatively safe environment where to rest and feed [13,46,50] and possibly to find shade during days with high temperatures (e.g. use of caves in Senegal [68] or to reduce thermoregulation costs [69]). The importance of the oil palm for food and nesting in chimpanzees has also been noted at other sites across West Africa [49–52].

We expected a difference between large and small human settlements when it comes to predicting chimpanzee presence; however, our model did not support this hypothesis, possibly because most of the villages in the study area are relatively small and many are isolated hamlets. Also, around settlements, farmers grow fruits which attract chimpanzees despite the risks associated with being detected and encountering people when foraging in orchards. In fact, we captured images of chimpanzees carrying domesticated fruits (i.e. mangos, pineapples) in four different cameras that were set near orchards close to human settlements.

We also hypothesised that chimpanzee relative abundance will be positively influenced by abandoned settlements because of the presence of domesticated fruit trees in these areas [40]. However, our model did not support this prediction. Although we recorded nests in these areas, chimpanzees may only visit these sites occasionally when fruits are available. Local people visit these areas to harvest available cultivated fruits and hunters also venture into these areas which attract wildlife such as monkeys and duikers (RMG pers. obs.). People’s continued usage of abandoned settlements, especially hunters, may act as deterrents to regular and more sustained chimpanzee frequentation of these areas.

The chimpanzees in our study area live in a challenging landscape where most of the trees are semi-domesticated oil palms growing across a changing agricultural landscape intermixed with swamps that act as a refuge for wildlife. The small settlements and the limited road network may be in the chimpanzees’ favour. However, their long-term future remains uncertain. Will chimpanzees still be present in this sort of landscape in the absence of the ubiquitous semi-domesticated oil palms, in which chimpanzees nest and feed, if the area were to be converted to oil palm plantations or another industrialised agricultural activity? What is the future of chimpanzees living in these degraded landscapes facing increased human population growth and development? How will chimpanzees cope with wider paved roads and larger settlements? The fact that more than half of the chimpanzee population in Sierra Leone is found in similar anthropogenic landscapes highlights the importance of our results. Conservation programmes should not neglect these chimpanzee populations if we want to secure their long-term survival, especially in areas where they are tolerated and hunting pressure on the species is low, or areas prone to conversion to industrialised activities and associated infrastructures. Further studies are also required to better understand chimpanzees’ ecological, demographic and social habits in similar anthropogenic habitats to inform chimpanzee responses to landscape changes. Such studies could help inform effective mitigation strategies aimed at improving people attitudes towards the species and balancing conservation efforts and development activities. There is also a growing need to understand what factors shape people’s tolerance of chimpanzees to increase initiatives meant at improving sustainable coexistence between people and chimpanzees. Successful protection measures should benefit people and chimpanzees alike. Conservation actions should focus on education and helping farmers to implement alternative agricultural methods to slash and burn farming and environmentally-friendly revenue generating activities. Such initiatives are critical to help preserve the habitat and key ecosystems services which people depend on and to improve the living standards of subsistence farmers. Such initiatives are expected to ameliorate people’s behaviour towards chimpanzees and their understanding of the role of the species in the landscape and the drivers behind crop foraging. One valuable approach may also be to develop agreements with farmers to allow strategic fallow areas to regenerate into community-managed forest refuges providing corridors for wildlife and vital natural resources and ecosystem services for both humans and wildlife.

## Supporting information

S1 Fig Trace and density estimate for each variable (roads and swamps) of the Markov Chain Monte Carlo (MCMC). (TIF)

S1 Table Dataset with trapping rates and number of events for chimpanzees and humans, including near distances in meters between camera locations and each variable, i.e. roads, settlements, abandoned settlements, mangroves, swamps and farmland. (XLSX)
