# Supplementary figures and images for: Factors influencing wild chimpanzee (Pan troglodytes verus) relative abundance in an agriculture-swamp matrix outside protected areas
Source: PLoS One. 2019 May 16;14(5):e0215545. doi: 10.1371/journal.pone.0215545 (PMC6522039; doi:10.1371/journal.pone.0215545)

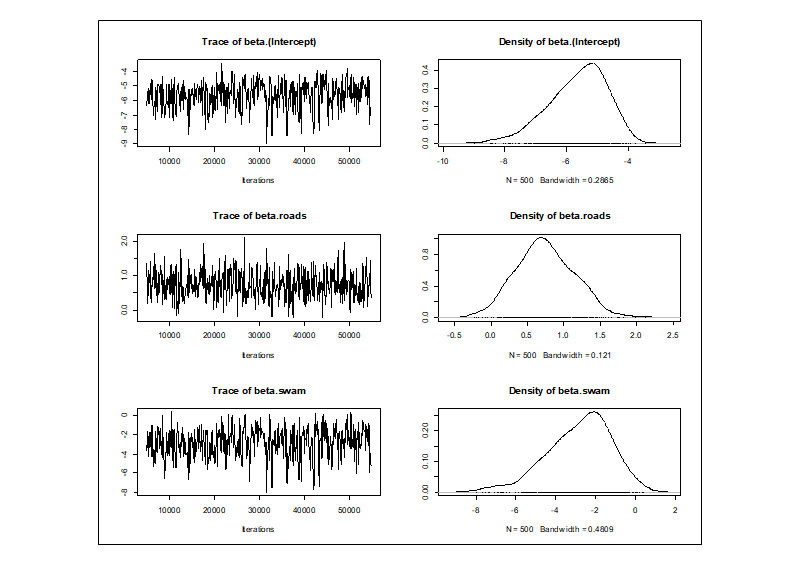

Supplement: S1 Fig — (TIF) [file pone.0215545.s001.tif]
